# Trastuzumab Emtansine (T-DM1) and stereotactic radiation in the management of HER2+ breast cancer brain metastases

**DOI:** 10.1186/s12885-021-07971-w

**Published:** 2021-03-04

**Authors:** Matthew N. Mills, Chelsea Walker, Chetna Thawani, Afrin Naz, Nicholas B. Figura, Sergiy Kushchayev, Arnold Etame, Hsiang-Hsuan Michael Yu, Timothy J. Robinson, James Liu, Michael A. Vogelbaum, Peter A. Forsyth, Brian J. Czerniecki, Hatem H. Soliman, Hyo S. Han, Kamran A. Ahmed

**Affiliations:** 1 Departments of Radiation Oncology, H. Lee Moffitt Cancer Center and Research Institute, 12902 Magnolia Dr., Tampa, FL 33612 USA; 2 University of South Florida, Morsani College of Medicine, Tampa, FL 33612 USA; 3 Departments of Radiology, H. Lee Moffitt Cancer Center and Research Institute, Tampa, FL 33612 USA; 4 Departments of Neuro Oncology, H. Lee Moffitt Cancer Center and Research Institute, Tampa, FL 33612 USA; 5 Departments of Breast Oncology, H. Lee Moffitt Cancer Center and Research Institute, Tampa, FL 33612 USA

**Keywords:** Trastuzumab emtansine, Kadcyla, Breast cancer, Brain metastases, Stereotactic radiotherapy

## Abstract

**Background:**

Due to recent concerns about the toxicity of trastuzumab emtansine **(**T-DM1) with stereotactic radiation, we assessed our institutional outcomes treating HER2-positive breast cancer brain metastases (BCBM) with T-DM1 and stereotactic radiation.

**Methods:**

This is a single institution series of 16 patients with HER2-positive breast cancer who underwent 18 stereotactic sessions to 40 BCBM from 2013 to 2019 with T-DM1 delivered within 6 months. The Kaplan-Meier method was used to calculate overall survival (OS), local control (LC), distant intracranial control (DIC), and systemic progression-free survival (sPFS) from the date of SRS. A neuro-radiologist independently reviewed follow-up imaging.

**Results:**

One patient had invasive lobular carcinoma, and 15 patients had invasive ductal carcinoma. All cases were HER2-positive, while 10 were hormone receptor (HR) positive. Twenty-four lesions were treated with stereotactic radiosurgery (SRS) to a median dose of 21 Gy (14–24 Gy). Sixteen lesions were treated with fractionated stereotactic radiation (FSRT) with a median dose of 25 Gy (20-30Gy) delivered in 3 to 5 fractions. Stereotactic radiation was delivered concurrently with T-DM1 in 19 lesions (48%). Median follow up time was 13.2 months from stereotactic radiation. The 1-year LC, DIC, sPFS, and OS were 75, 50, 30, and 67%, respectively. There was 1 case of leptomeningeal progression and 1 case (3%) of symptomatic radionecrosis.

**Conclusions:**

We demonstrate that stereotactic radiation and T-DM1 is well-tolerated and effective for patients with HER2-positive BCBM. An increased risk for symptomatic radiation necrosis was not noted in our series.

## Background

HER2 overexpression is a significant risk factor for the development of breast cancer brain metastases (BCBM) [1]. Although an uncommon site of first relapse, eventually 30–55% of HER2+ breast cancers will develop central nervous system (CNS) metastasis [2]. However, given improved systemic therapy options, patients with HER2+ breast cancers have an improved prognosis over HER2 negative BCBMs, with a median survival of over 20 months in good performance status patients [3].

Radiation therapy is a cornerstone in the management of BCBM [1]. Combination therapy with radiation and systemic therapy allows for potential synergistic benefit [4] at the risk of increased toxicity. Given the concurrent disease burden and the role of systemic therapy, it is important to note clinically significant rates of toxicity with combined therapy. One of the most worrisome late side effects of CNS radiation therapy is radionecrosis. The mechanism for radionecrosis is unclear but may be through vascular injury, hypoxia, injury to oligodendrocytes, and chronic inflammation in response to these injuries [5]. VEGF secretion also might play a role in radionecrosis, with bevacizumab occasionally utilized as therapeutic intervention for radionecrosis [6].

Given the results of the KATHERINE study which revealed improvements in invasive disease-free survival with the receipt of adjuvant trastuzumab emtansine (T-DM1) over trastuzumab alone in patients with residual disease following neoadjuvant chemotherapy and surgical resection [7], T-DM1 is increasingly being prescribed to early stage HER2 positive breast cancer patients [8]. A recent study from Stumpf et al. noted clinically significant rates of radionecrosis with the receipt of T-DM1 and stereotactic radiation with the hypothesis that mediation is through upregulation of aquaporin-4 [9]. The rates of radionecrosis were 39% in 23 patients receiving T-DM1 and 5% in those that did not. Given the high rates of radionecrosis noted with T-DM1 and stereotactic radiation, we assessed our experience in the management of HER2+ brain metastases with T-DM1 and stereotactic radiation.

## Methods

Patients with HER2+ BCBM who received stereotactic radiosurgery (SRS) or fractionated stereotactic radiotherapy (FSRT) from December 2013 to December 2019 were identified in a prospectively managed database. Patients were included if they were treated with stereotactic radiation within 6 months of receiving T-DM1 (either before, during, or after administration), as previously reported [10–12]. The study was approved by the University of South Florida Institutional Review Board.

### Stereotactic radiation technique

Brain metastases were evaluated with magnetic resonance imaging (MRI) (Siemens Sonata, Siemens Medical Systems, Erlangen, Germany) with 1 mm slices for prior to the delivery of radiation. The MRI image was co-registered and fused with computed tomography (CT) simulation imaging (General Electric Medical System, Milwaukee, WI). Patient immobilization was accomplished with a head mask fixation system (BrainlabAG, Feldkirchen, Germany), as previously described [11]. Treatments were delivered using multiple dynamic conformal arcs or intensity modulated radiotherapy. Image guidance was provided with the BrainLab ExacTrac positioning system (BrainlabAG, Feldkirchen, Germany). The planning target volume (PTV) was generated using a uniform 1–2 mm expansion of the gross tumor volume (GTV). Patients were treated in a single session in 24 (60%) of lesions and in multiple treatment fractions in 16 (40%) of lesions. Six lesions (15%) underwent prior surgery. Doses were prescribed to ensure coverage of at least 95% of the PTV with the prescription dose.

### Follow-up

Patients were assessed by the treating radiation oncologist, neurosurgeon, and/or medical oncologist with MRI imaging at 2–3 month intervals with neurological clinic exams and MRI. Local brain metastasis failure was defined by RANO-BM criteria [13] that remained consistent or demonstrated continued progression on subsequent imaging, whereas local brain metastases control (LC) included all treated lesions not meeting this definition. Radionecrosis was considered an increase in size of peripheral enhancement of the lesion on T1 weighted (WI) contrast enhancement imaging with the development of the central necrotic area, haziness of the borders and enlarging peripheral edema; 2) significant regression (> 50%) or stability of the lesions for > 3 months without additional treatment; 3) evidence of intralesional hemorrhage involving the entire lesion identified as susceptibility weighted imaging (SWI) of T1 WI without enlargement; or 4) focal area of hypoperfusion on dynamic susceptibility contrast (DSC) perfusion MRI.

Distant brain metastases failure was defined as new brain metastases or leptomeningeal enhancement outside the previously irradiated field. Distant intracranial control (DIC) was defined as freedom from development of brain metastases or leptomeningeal disease outside of the irradiated field. Imaging was independently reviewed by a neuro-radiologist (SK).

### Statistical analysis

Statistical analyses were performed using JMP 13 (SAS Institute Inc., Cary, NC, USA). Descriptive statistics were used to summarize the cohort. The LC and DIC were estimated from the date of radiation treatment, while overall survival (OS) was calculated both from the date of stereotactic radiation and the date of BCBM diagnosis to the date of death. The Kaplan-Meier (KM) method was used to estimate treatment outcomes, with the log-rank test used to test differences between groups.

## Results

### Patient and treatment characteristics

Patient and treatment characteristics are detailed in Table 1. A total of 16 patients treated over 18 treatment sessions to 40 HER2+ BCBM lesions were identified. There were 15 patients diagnosed with invasive ductal carcinoma and 1 patient with invasive lobular carcinoma. Ten patients (63%) were originally diagnosed with de novo metastatic breast cancer. Median follow-up from the date of stereotactic radiation was 13.2 months (range: 0.1–55.5 months) and median follow-up from the date of brain metastases diagnosis was 68.6 months (16.5–249 months). Breast cancer subtypes were 63% (*n* = 10) HR+/HER2+ and 38% (*n* = 6) HR−/HER2 + .

**Table 1:** Patient and Treatment Characteristics

Variable	n	%
**No. of Patients**	16	
**Treatment Sessions**	18	
**No. of Lesions**	40	
**F/U from RT (months)**		
Median (range)	13.2 (0.1–55.5)	
**F/U from Brain Metastases Diagnosis (months)**		
Median (range)	68.6 (16.5–249)	
**Age at time of RT**		
Median (range)	56 (40–85)	
**KPS**		
100	2	13%
90	10	63%
80	4	25%
**Lesions Treated Per Patient**		
Median (range)	2 (1–6)	
**Receptors**		
HR+/HER2+	10	63%
HR−/HER2+	6	38%
**Concurrent Therapy with SRS**		
None	9	50%
Chemotherapy	1	5%
TP	2	11%
Chemotherapy + TP	2	11%
ET	2	11%
ET + TKI + TP	1	5%
ET + TP	1	5%

Abbreviations: F/U = follow up, RT = radiation therapy, KPS = Karnofsky performance status, TKI = tyrosine kinase inhibitor, ET = endocrine therapy, TP = trastuzumab and/or pertuzumab, HR = hormone receptor, SRS = stereotactic radiosurgery

Radiation details are described in Table 2. The median PTV of lesions was 0.92 cm^3^ (range: 0.08–66.2 cm^3^). The median dose of SRS was 21 Gy (range: 14–24 Gy) treated in a single fraction, and for lesions treated with FSRT was 25 Gy (20–30 Gy) in a median of 5 fractions (range: 3–5). Six lesions (15%) were treated post-operatively. Stereotactic radiation was delivered concurrently with T-DM1 in roughly half of the treated lesions (*n* = 19; 48%). Stereotactic radiation was delivered before or after T-DM1 in 28% (*n* = 11) and 25% (*n* = 10) lesions, respectively. In patients not treated concurrently, the median time between receipt of T-DM1 and stereotactic radiation was 2.7 months (range: 1.7–6 months).

**Table 2:** Radiation Treatment Details

Variable	n	%
**Technique**		
SRS	24	60%
FSRT	16	40%
**SRS Dose (Gy)**		
Median (range)	21 (14–24)	
**FSRT Dose (Gy)**		
Median (range)	25 (20–30)	
Fractions	5 (3–5)	
**Postop**	6	15%
**PTV (cm**^**3**^**)**		
Median (range)	0.92 (0.08–66.2)	
**RT in Relation to T-DM1**		
Before	11	28%
After	10	25%
Concurrent	19	48%

Abbreviations: PTV = planning target volume, SRS = stereotactic radiosurgery, FSRT = fractionated stereotactic radiotherapy

### Clinical outcomes

**Fig. 1:**
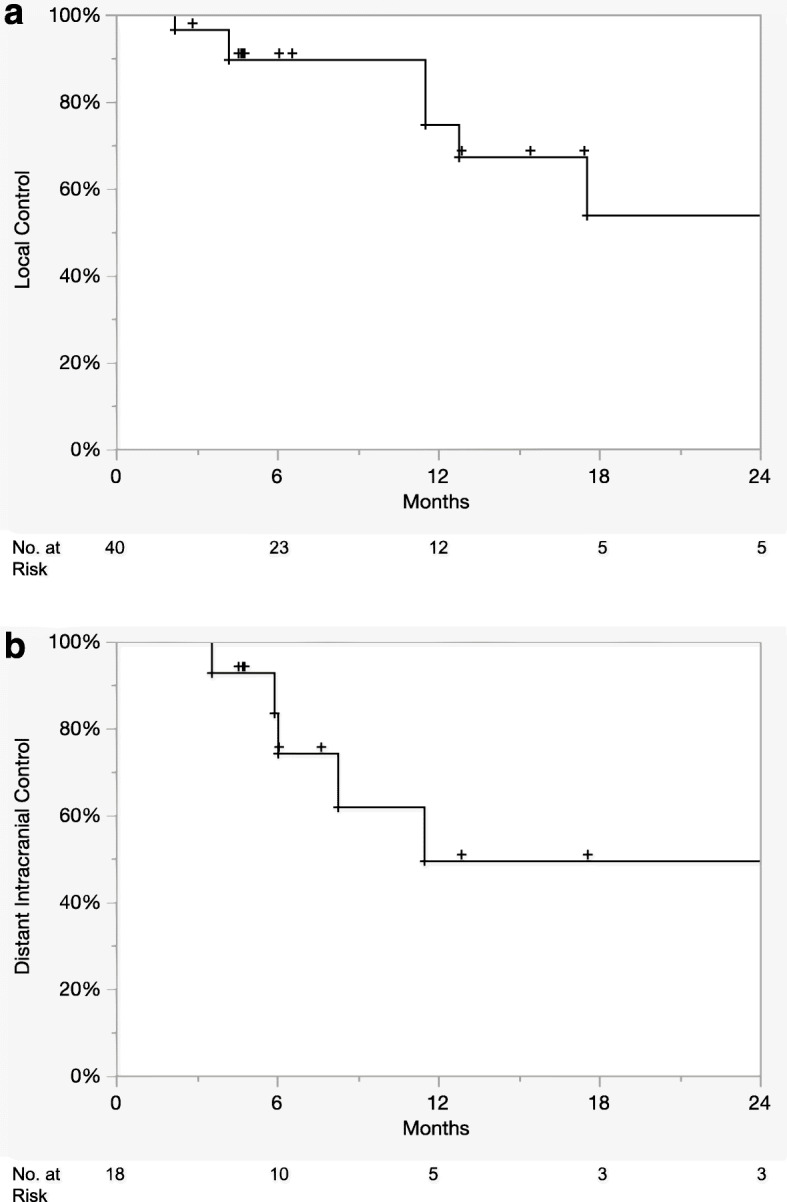
Kaplan-Meier **a**) local control and **b**) distant control following stereotactic radiation

**Fig. 2:**
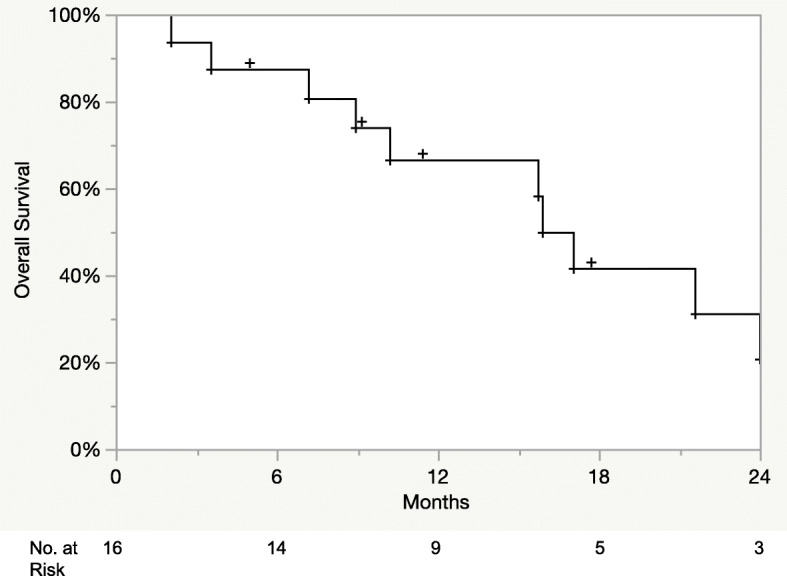
Kaplan-Meier overall survival following stereotactic radiation

Twelve- month LC and DIC was 75 and 50%, respectively (Fig. 1a and b). One patient who underwent post-operative FSRT eventually developed leptomeningeal disease at 15 months post SRS. Median OS was 15.9 months (95% CI 9–24 months) from the date of stereotactic radiation and 26.6 months (95% CI 12–56 months) from the date of brain metastases diagnosis. Extracranial systemic control at 12 months was 30%. Twelve-month OS was 67% following stereotactic radiation (Fig. 2) and 79% following brain metastases diagnosis, respectively.

### Toxicity

One case of radionecrosis was noted (3%). This patient was admitted to the hospital with worsening neurologic symptoms, including aphasia and confusion, 179 days after receiving SRS to 5 brain lesions and FSRT to 1 brain lesion, with concurrent T-DM1 (first dose given 8 days prior to stereotactic radiation). MRI of the brain demonstrated an increase in size, enhancement, and surrounding vasogenic edema of a previously treated right temporal lobe lesion, consistent with radionecrosis, as well as a new brainstem metastatic lesion. She received intravenous steroids, followed by intravenous bevacizumab, which improved her symptoms slightly.

Mild radiation-related symptoms were noted during 8 treatment sessions (45%) including grade 1–2 headaches and fatigue. One instance of grade 2 headache occurred and resolved completely following treatment with steroids. A total of 8 patients were administered steroids during the course of radiation: one prophylactically and seven therapeutically due to symptoms attributed to intracranial metastasis. No other unanticipated side effects were noted.

## Discussion

We report our single-institution experience in the management of HER2+ BCBM treated with stereotactic radiation and T-DM1. One case of radionecrosis was noted in our experience with no other unexpected neurologic toxicities similar to what would be expected with receipt of stereotactic radiation alone. In addition, we note adequate local control rates and promising rates of survival after SRS, consistent with experiences with stereotactic radiation alone [14].

Trastuzumab emtansine is an antibody-drug conjugate of trastuzumab and emtansine, a microtubule inhibitor [8, 15]. This conjugate allows for the intracellular delivery of emtansine to HER2 overexpressing cells via receptor-mediated endocytosis [16]. Proteolytic degradation of the antibody leads to release of emtansine, which inhibits microtubule assembly, causing mitotic arrest and apoptosis. The utilization of T-DM1 in patients with HER2+ breast cancer has recently increased, as clinical trials have demonstrated T-DM1 to be a well-tolerated and effective treatment for patients with HER2+ breast cancer with residual disease after neoadjuvant treatment [17] and in patients with HER2+ advanced or metastatic breast cancer [7, 18]. In fact, a retrospective subset analysis of the EMILIA trial included 95 patients with asymptomatic baseline CNS metastatic disease and demonstrated improved OS in patients treated with T-DM1 in comparison to lapatinib and capecitabine [19]. Recent studies have demonstrated that patients with HER2+ BCBM treated with SRS have improved survival when compared to HER2- patients, likely due to the improved systemic control with HER2-targeted agents such as T-DM1 [14, 20].

Studies have demonstrated the ability of T-DM1 to penetrate the altered blood-tumor barrier (BTB). In a murine model of HER2+ breast cancer, Askoxylakis et al. found that T-DM1 delayed the growth of BCBM and increased survival in comparison to mice that received trastuzumab [21]. Patients previously treated with radiation likely experience a higher degree of T-DM1 penetration, as multiple studies have found that WBRT or SRS can increase the permeability of the BTB [4, 22]. Indeed, several recent small series of patients with HER2+ BCBM have demonstrated T-DM1 to be an effective treatment, even in patients with treatment refractory brain metastases [23–26]. There have even been case reports of long-term treatment response for patients with leptomeningeal disease treated with radiation and T-DM1 [27, 28].

Radionecrosis is the most significant late toxicity of SRS alone, with an estimated incidence of roughly 7% after SRS [29]. Per RTOG 9005, the rates of radionecrosis of tissue reirradiated with single fraction radiosurgery were 8% at 12 months and 11% at 24 months [30]. Concurrent systemic therapies may increase the risk for this toxicity, as there have been reported increased rates of radionecrosis after stereotactic radiation with concurrent immune checkpoint inhibitors [31]. However, other studies were unable to demonstrate an increased risk after combination therapy [11, 32–34]. Similarly, there is conflicting evidence as to whether concurrent stereotactic radiation with BRAF inhibition increases the risk of radiation necrosis [35, 36]. Recent evidence suggests that SRS with concurrent lapatinib, another HER2-targeted agent, did not increase the risk for radiation necrosis [37, 38].

There is concern that the combination of SRS and T-DM1 may elevate the risk of radiation necrosis, as there have been recent case reports of pathology-confirmed radiation necrosis in patients treated with SRS followed by T-DM1 [39, 40]. In a series of 12 patients with BCBM treated with concurrent (*n* = 4) or sequential (*n* = 8) SRS and T-DM1, Geraud et al. reported 4 cases of radiation necrosis (33.3% of treatments), with a higher incidence in patients treated concurrently [41]. In another series of 45 patients with BCBM treated with SRS, Stumpf et al. found a significantly higher rate of symptomatic radiation necrosis in patients who received T-DM1 (9/23 patients) compared with those who did not (1/22 patients) [9]. Of the 9 patients who were found to have radionecrosis in the T-DM1 group, 4 patients received sequential SRS and 5 were treated concurrently. Though exact mechanisms for the role of T-DM1 in radiation necrosis is ill-defined, several theories have been postulated. Stumpf et al. demonstrated that T-DM1 causes an upregulation of aquaporin 4 and enhanced cytotoxic effects of radiation on reactive astrocytes, which contribute to astrocytic swelling [9].

The rates of radiation necrosis in studies from Stumpf [9] and Geraud et al. [41] are considerably higher than in the present study (1/18 treatment sessions), and several important differences amongst the studies likely contributed to these incongruent results. Radiation necrosis is challenging to accurately diagnose, as imaging characteristics so often mimic tumor progression. While the prior studies have defined clinically significant radiation necrosis as neurologic symptoms requiring hospitalization and treatment [9], the present study is the first series to utilize independent imaging review with a neuro-radiologist to aid radiation necrosis diagnosis. Importantly, these series represent relatively small sample sizes with significant heterogeneity in patient and treatment characteristics. In addition, while the range between delivery of SRS and receipt of T-DM1 was as long as 1426 days in the Stumpf study [9], there was a smaller maximum range of 6 months used to select patients in the current study. The half-life of T-DM1 is approximately 4 days [42] and thus longer time intervals from radiation and receipt of T-DM1 may not accurately reflect toxicity from combined treatment. Finally, the extended survival of patients with HER2+ disease treated with T-DM1 may have contributed to a higher observed incidence of late toxicity in these patients who received a number of other systemic treatments.

The present study has several important limitations, including its retrospective nature, heterogeneous patient cohort, and small sample size. In addition, the relatively short median follow up is a significant limitation due to the protracted survival of patients with HER2-positive BCBM. As pathologic confirmation was unavailable, the diagnosis of radiation necrosis depended upon careful review of the follow up imaging by a single neuro-radiologist.

## Conclusions

In conclusion, despite the small sample size, we show the combination of T-DM1 with stereotactic radiation to be well-tolerated, with a similar control rate and toxicity profile to stereotactic radiation alone. These results highlight the need for prospective investigation to definitively characterize the toxicity of combined treatment with SRS and T-DM1.

## Data Availability

The datasets used and/or analysed during the current study are available from the corresponding author on reasonable request.

## Abbreviations

BCBM: breast cancer brain metastasis
CNS: central nervous system
T-DM1: emtansine trastuzumab
SRS: stereotactic radiosurgery
FSRT: fractionated stereotactic radiotherapy
MRI: magnetic resonance imaging
CT: computed tomography
GTV: gross tumor volume
PTV: planning target volume
LC: local control
WI: T1 weighted contrast
SWI: susceptibility weighted imaging
DSC: dynamic susceptibility contrast
DIC: distant intracranial control
OS: overall survival
KM: Kaplan-Meier
BTB: brain-tumor barrier
